# Teacher and Peer Responses to Warning Behavior in 11 School Shooting Cases in Germany

**DOI:** 10.3389/fpsyg.2020.01592

**Published:** 2020-07-24

**Authors:** Nora Fiedler, Friederike Sommer, Vincenz Leuschner, Nadine Ahlig, Kristin Göbel, Herbert Scheithauer

**Affiliations:** ^1^Department of Education and Psychology, Freie Universität Berlin, Berlin, Germany; ^2^Department of Police and Security Management, Berlin School of Economics and Law, Berlin, Germany

**Keywords:** warning behaviors, threat assessment, targeted school violence, school shooting, case study

## Abstract

Warning behavior prior to an act of severe targeted school violence was often not recognized by peers and school staff. With regard to preventive efforts, we attempted to identify barriers to information exchange in German schools and understand mechanisms that influenced the recognition, evaluation, and reporting of warning behavior through a teacher or peer. Our analysis is based on inquiry files from 11 cases of German school shootings that were obtained during the 3-year research project “Incident and case analysis of highly expressive targeted violence (TARGET).” We conducted a qualitative retrospective case study to analyze witness reports from school staff and peers. Our results point to subjective explanations used by teachers and peers toward conspicuous behavior (e.g., situational framing and typical adolescent behavior), as well as reassuring factors that indicated harmlessness (e.g., no access to a weapon). Additionally, we found organizational barriers similar to those described in US-American case studies (e.g., organizational deviance).

## Introduction

A key finding from the retrospective analysis of cases of severe targeted school violence (e.g., school shootings) is that these violent acts can be regarded as an endpoint of a long-term negative developmental pathway (e.g., Levin and Madfis, 2009; Scheithauer et al., 2014; Sommer et al., 2020). Furthermore, there is a consensus in the literature on school shootings that several types of warning behaviors and indicators of a personal crisis were observable by persons in the school, family, or peer context prior to a violent act (Hoffmann et al., 2009; Rocque, 2012; Bondü and Scheithauer, 2014b; Gerard et al., 2016). Perpetrators either gave warning to peers by announcing an attack (“leakage” cf. Meloy et al., 2012), made direct threats to kill toward potential victims, or displayed behaviors that can be regarded as indicative of a psychosocial crisis (e.g., sudden behavioral changes, social withdrawal, or school absenteeism). However, as international case studies reveal, warning behaviors were often not recognized or responded to by the perpetrators’ peers and members of school staff (Daniels J.A. et al., 2007; Syvertsen et al., 2009; Scheithauer and Bondü, 2011). Newman et al. (2004) found the inability of the social support systems to identify and bundle warning behaviors—beside social marginality, individual vulnerabilities, access to guns, and cultural scripts—to be one of five necessary conditions for school shootings. It is significant to understand why warning behaviors that became apparent in the school context were not recognized or adequately identified by peers and school staff, and—if identified—a progression of a negative psychosocial development could not be averted through case management measures. In that respect, research findings from US-American case studies provide some explanations taking schools’ organizational structures, as well as peer group norms, into account (Fox and Harding, 2005; Daniels J. et al., 2007; Pollack et al., 2008). In the following, we present structural barriers as well as challenges school staff and peers are facing when identifying, assessing, evaluating, and handling students’ warning behaviors.

### Awareness of Potential Warning Behavior

Research provides evidence that a school’s organizational complexity and lack of resources are barriers to successful communication and crisis management. Fox and Harding (2005) consider “organizational deviance” as a structural obstacle, meaning that warning behavior of students is not recognized and properly handled by school staff and results from routines and behaviors in an institution that were established to serve a specific purpose. For instance, Fox and Harding (2005) found a general tendency in members of large organizations to primarily respond to behavior that disturbs an organization’s day-to-day functioning (e.g., aggressive behavior; “The squeaky wheel gets the oil”; “decoy problem”). However, this scheme of action may not be sufficient in cases of students’ trajectories leading to severe school violence. Many school shooters did not show aggression or apparent frustration prior to an attack, even while already engaging into planning behavior, but were more “invisible kids,” a term used by Bender et al. (2001) referring to students who did not draw much teacher attention on them. Moreover, a single school staff member’s autonomy in day-to-day decision-making and task segregation can result in “structural secrecy” and “loose coupling” leading to what is described as “institutional memory loss” in the literature (Vossekuil et al., 2002; Fox and Harding, 2005). Due to a lack of time resources, information on student warning behavior is often not shared with colleagues or reported to authorities (Harding et al., 2002). Instead, an observation remains fragmented within a school. Moreover, typical conflict situations in a school require quick reactions from educational staff who primarily rely on gut feelings instead of an informed decision-making procedure (Leuschner et al., 2011). A pilot study conducted in 2009, the Berlin Leaking Project, indicated a significant lack of knowledge and uncertainty in risk assessment among German school staff (Bondü et al., 2011). Teachers reported a strong need for general sensitization and intense expert training to create awareness for the topic. Additionally, participants have demanded increasing support and counseling from their local professional network (Leuschner et al., 2011). “Information fragmentation” can also occur when observations of a student behavior are not exchanged with professionals from a school’s external network and local service institutions. Eventually, a lack of inter-institutional cooperation can become a significant barrier to effective case management and the initialization of supportive measures for an adolescent in crisis (Harding et al., 2002). Finally, an insufficient documentation of observations is another structural risk factor: data on apparently harmless disciplinary incidents are often not recorded in a student’s file due to law restrictions or a well-meant “clean-slate” mentality. Information on a student’s social biography, family background, or psychological particularities literally “diffuses” and cannot be integrated in case assessment after a student transitions to secondary school or another school district. From a developmental perspective, this can have a harmful impact, since teachers at a new school will have difficulties to evaluate the progression of a student’s crisis properly.

### Identification and Correct Interpretation of Students’ Potential Warning Behavior

In the majority of cases, peers—and not adults—were the first to identify behavioral changes (Bender et al., 2001; Oksanen et al., 2013; Madfis, 2014). In most cases, peers of subsequent perpetrators had advanced knowledge about a planned attack but followed a “code of silence” and opted to withhold knowledge or concern from an adult (Daniels J.A. et al., 2007; Syvertsen et al., 2009; Madfis, 2014). The “code of silence” is a behavioral norm followed by adolescents to protect a peer from trouble, implying not to share conspicuous information (e.g., leaking behavior, or a peer’s problems) with an adult or authority figure. Thus, for the majority of school shootings, indicators of a perpetrator’s negative psychosocial development were hardly visible to adults; consequently, school staff did not obtain the significant information necessary to identify a student in crisis. In a qualitative study conducted by the United States Secret Service as part of the United States Safe School Initiative (SSI), Pollack et al. (2008) interviewed 119 students that were involved as bystanders in school shootings that happened in the United States. The authors refer to bystanders as “students who had some prior knowledge that an attack was planned.” The study revealed that 59% of bystanders reported advanced knowledge about the perpetrator’s violent fantasies, often days or weeks prior to the attack, and 82% had their information directly from the perpetrator, but did not share it (Pollack et al., 2008). Additionally, school staff face challenges in the identification and correct interpretation of students’ warning behavior potentially leading to school shootings. Predictions of school shooting behavior based on risk factors of former shooters using checklists (i.e., profiling) are inappropriate, as they would lead to a high amount of falsely identified students, which may result in stigmatization and unreasonable reduced sense of safety in schools (Borum et al., 2010). School staff may tend to rely on personal presumptions or media-disseminated knowledge about school shootings without reliable information about students’ warning behavior.

### Evaluation of Seriousness of Students’ Threats and Warning Behavior

Peer bystanders mostly underestimated the seriousness of a threat (e.g., threat was a “joke” or made “in jest”) or did not believe their peer would be able to carry it out. Pollack et al. (2008) found “misjudgment of the likelihood and immediacy of an attack” and “disbelief in seriousness of threats” as explanations to keep information to themselves. Finally, Wike and Fraser (2009) identified “high-risk-school cultures” among US-American schools with a school shooting attack, which described a social climate that encourages low school bonding and high “social stratification” and provides few opportunities for participation, rewards, and positive interaction between teachers and students, hence fostering bullying, harassment, and other forms of violence. On the contrary, Eliot et al. (2010) used a sample of 7,318 students from 291 schools from the Virginia High School Safety Study to examine the correlation of characteristics related to school culture with school. The authors found that the students’ willingness to seek help from an adult when confronted with a threat of violence increases with a supportive school climate and perceived support from teachers, as well as a positive attitude toward the school (Eliot et al., 2010).

### Finding an Appropriate Response to Students’ Warning Behavior

One effort in preventing school violence in the United States is described under the term “zero tolerance,” referring to a range of policies that seek to impose severe sanctions (e.g., suspensions and school expulsion) for minor offenses in hopes of preventing more serious ones (Borum et al., 2010; Muschert and Madfis, 2013). However, due to the lack of empirical evidence of any positive effect in deterring or reducing school violence, zero-tolerance policies have been questioned and even criticized as measures contrary to the principles of a healthy child development (Gregory and Cornell, 2009; Borum et al., 2010). The challenge of finding an appropriate response to students’ warning behavior can be illustrated by Sommer et al. (2016) who analyzed interventions of school staff when confronted with a student’s psychosocial crisis at risk for a school shooting (cf. Bondü and Scheithauer, 2014c). While in most cases school staff responded to the student crisis or warning behavior by initiating resource-oriented measures, finding appropriate interventions in high-risk cases (e.g., student in possession of guns, detailed execution plans) proved to be a particular challenge. Often lacking sustainable knowledge or networks to accessible experts (e.g., prevention officers and psychotherapists), school staff mostly dealt with the students’ critical behavior within the institution, which might have resulted in feelings of overstraining and unsafety (Sommer et al., 2016).

To summarize, a growing body of case studies and research on organizational risk factors has produced valuable insights into the phenomenon of severe, targeted school violence from a social framing perspective. The identification of social and structural risk factors, organizational deviance, and a negative school climate along with a better understanding of why peers of subsequent perpetrators underestimated the seriousness of threats points to opportunities of school-wide prevention and measures with a focus on the individual perpetrator (e.g., risk assessment). The purpose of this study is to identify barriers to information exchange in German schools with a school shooting incident and to highlight organizational risk factors as well as risk factors resulting from peer group norms. Additionally, the paper will discuss underlying mechanisms and individual assumptions of peers and teachers that had an impact on the identification of conspicuous behavior and to investigate them more closely. With regard to preventive efforts, the following research questions will be addressed: (1) Which measures of case management were initiated either within the schools, or with the help of a school’s professional support network? (2) How did peers respond to threats and leakage, and what can we learn about adolescent code of silence and peer evaluation of conspicuous behavior? (3) Which assumptions and specific factors can be found in the material that led teachers and peers to assess a conspicuous behavior as concerning or alarming? How did teachers and peers attempt to explain a behavioral change in the perpetrator that in retrospect can be considered a warning behavior?

Eventually, by integrating research findings from United States studies on school risk factors with results from the analysis of German school shooting cases, we aim to introduce an environmental perspective—in addition to the identification of individual risk factors—on the developmental pathways toward school shootings. A study on motives and specific constellations of individual risk factors of the perpetrator (e.g., mental disorders) provides an understanding of why an individual commits a violent act. In addition, a deeper investigation of social and organizational risk factors will help to explain why warning signs were not taken seriously, or individual support measures failed, which could eventually open new windows for prevention (e.g., by enhancing the expertise of persons within the social environment of an adolescent in crisis, such as school staff and peers) and simultaneously increase their feelings of safety. Overall, the study serves the purpose to bring light to the question why past acts of severe targeted violence could not be averted by school-related interventions.

## Materials and Methods

### Case Selection

Based on a definition from Bondü and Scheithauer (2014a, b) of school shootings, the following inclusion criteria to select a case were applied: (1) violent act was planned and executed by a current or former student of the school; (2) potentially lethal weapons were used (including non-shooting weapons, such as bombs or large knives); (3) the perpetrator had an intention to kill multiple victims associated with the school context; and (4) the perpetrator was not older than 25 years old when committing the violent act. Using the GENIOS and LexisNexis media databases, online archives of a total of 340 daily and weekly newspapers were systematically screened for the following search terms: “Amok (rampage),” “School Shooting,” “Terroranschlag (terroristic attack),” and “Mordanschlag (assassination attempt).” Through this search, 46 cases of potentially lethal school violence were identified, with a total of 11 meeting our inclusion criteria. The inquiry files from those 11 cases of German school shootings were obtained from the law enforcement authorities by the TARGET Research Group during the project “Incident and case analysis of highly expressive targeted violence (TARGET)” between 2013 and 2016. The average age of the perpetrators ranged from 13 to 23 years (*M* = 17.5, *SD* = 2.8); two were females and five committed suicide after the attack. Table 1 summarizes all cases that were included in the analysis. To preserve the anonymity of the perpetrators and follow strict data protection guidelines according to German law, the perpetrators’ names will neither be published in the following sections, nor will the material analyzed and reported allow for their identification. We used inquiry files instead of media reports to avoid bias by subjective theories of journalists (cf. Danner and Carmody, 2001). The obtained files included court reports, judgments (if the perpetrator did not commit suicide following the shooting), comprehensive police investigative, as well as witness reports, forensic-psychiatric expert assessments, and additional personal documents written by the perpetrator (e.g., diaries, unpublished testimonies, or personal essays).

**TABLE 1:** Cases of school shootings in Germany used for qualitative analysis.

**Time, place**	**Course of offense**
03/19/2002, Brannenburg	A 16-year-old student shoots his teacher with one of his father’s guns. He himself survives badly injured after attempting suicide.
02/19/2002, Eching/Freising	After killing two of his co-workers, a 21-year-old former student fatally wounds the headmaster of his former school and seriously injuries another teacher. He then commits suicide.
04/26/2002, Erfurt	A 19-year-old former student kills 12 teachers, 2 students, 1 administrative employee, and 1 police officer at his former school with a gun before shooting himself.
07/02/2003, Coburg	A 16-year-old student shoots at his classroom teacher. However, he misses her twice. A second teacher who entered the classroom is wounded by a bullet, before the offender takes a fellow student hostage and finally shoots himself.
11/20/2006, Emsdetten	An 18-year-old student injures 36 persons at his former school with firearms and smoke bombs, before committing suicide.
03/11/2009, Winnenden	The shooting of a 17-year-old former student resulted in the deaths of 12 people at his former school and 3 civilians he killed at a car dealership. His flight lasted several hours. He committed suicide, when he was surrounded by police forces.
05/11/2009, St. Augustin	A 16-year-old girl planned to stab several teachers and students at her school and to set the school on fire, using Molotov cocktails. When she was detected by a classmate, she injured her and fled from school. She turns herself over to the police in the evening of the same day.
09/17/2009, Ansbach	An 18-year-old perpetrator armed with Molotov cocktails and an axe injured 15 people, 2 of them severely. Police arrived on the scene shortly after the offense began and took him into custody.
02/18/2010, Ludwigshafen	A 23-year-old former student armed with a knife and a starter pistol stabbed his former teacher at his school to death. He was arrested shortly after.
11/09/2011, Ballenstedt and 02/26/2013, Wernigerode	Armed with several knifes and an axe, a 13-year-old girl sets a fire in the school’s hallway and threatens to kill classmates. Before injuring somebody she is arrested by police. One and a half year after the first event and a stay at a psychiatric clinic, she attacks her classmates at her new school using her father’s gas gun.
05/22/2012, Memmingen	A 15-year-old student enters his school with his father’s gun, aiming to shoot his former girlfriend. Since he cannot find her, he leaves school and starts shooting on a sports ground, where he was arrested by police.

### Qualitative Analysis

We conducted a qualitative retrospective case study mainly based on a content analysis approach (Kohlbacher, 2006) to analyze witness reports. Statements from school officials, teachers, friends of the perpetrator, and other peers were obtained during the police investigation. Information included warning behaviors and crisis symptoms that were recognized prior to the attack, subjective explanations for behavioral changes of the perpetrator, assumptions about the perpetrator’s motivation to commit a shooting, and detailed reports about the implemented measures once a conspicuous behavior was observed and which information was exchanged with others. We used the ATLAS.ti software, version 7.5 to develop our coding scheme. The coding procedure was initialized by an open coding of the first randomly selected case and was refined and enhanced based on the material of three additional cases. The analysis was mainly performed by one investigator (a female psychologist), who worked independently at the beginning of the thematic analysis. The coded material was continuously discussed with two other colleagues (one male sociologist and one female psychologist) to reach consensus on the main concepts. To assess the credibility of codes and themes, the entire research process was recorded, reviewed, and reflected upon by the investigators and discussed with three other members of the research team.

During that stage, the development of our coding scheme was shaped by an inductive approach with openness to find new meaning in the data. Data collection and analysis were developed together in an iterative process. The concepts emerging from the data were then integrated with preliminary theory-informed categories (e.g., barriers to effective identification of risk factors, warning behaviors) allowing us to generate more specific hypotheses and develop explanatory codes. Thus, all data have been organized around certain topics (e.g., code of silence), key themes, or central questions. To generate those categories, we integrated existing theories and findings from the literature on school shootings, as well as results from the sociological study of barriers to effective information exchange in schools. The first four cases, as well as all remaining cases, were then coded with the developed final coding scheme. The coding scheme reflecting theoretical constructs was refined by clustering open codes together into categories that were guided by theoretical concepts but simultaneously grounded on the empirical data. It includes definitions for all codes, categories (e.g., warning behaviors, or seriousness assessment factors) and sub-categories (e.g., violence-specific warning behavior, general crisis symptoms, or “alarming” and “reassuring” factors), as well as examples when they were applied. We examined the subjective explanations from school staff and peers once they became aware of a conspicuous behavior drawing on the retrospective analysis of witness reports. This was followed by an in-depth analysis of why a certain behavior was not adequately identified as warning behavior, and why the respective observation was not shared or reported. Additionally, we obtained information on the frequency of case management measures that were initiated by the schools and their professional network, as well as distinguished specific responses from peers that had observed leakage or a threat, or had other knowledge that a school shooting was planned.

## Results

### Research Question 1: Measures of Case Management and Student Support

Case management measures were divided into measures that were initiated within a school drawing on the expertise and the professional background of school staff and related professions (within institution), and measures that were implemented within the larger professional network of a school. These required information exchange and cooperation between local institutions (e.g., a school psychologist, youth counselor, and police) or the student’s family (between institutions). Table 2 reports the frequencies of measures that were initiated along the developmental pathway in the analyzed cases.

**TABLE 2:** Measures of case management in school shooting cases in Germany.

**Measures for case management**	**Number of cases**
Professional network cooperation (between institutions):	
Talk to parents at least once (initiated by parents or school)	11
Refer student to counseling, diagnostic investigation, or therapy	7
Temporary hospitalization (crisis intervention or long-term therapy)	6
Involve police	4
Involve local school psychologist Measures within the school (inside institution):	3
Talk to student in crisis	11
Pedagogical reaction	10
Information exchange with a colleague	8
Regulatory measure	7
Expulsion from school or vocational training	4
Involve school counselor	2
Crisis prevention (structured or guideline-informed)	2

#### Measures Within the School

In all 11 cases, a school official or teacher at least once initiated some type of supportive face-to-face conversation with the student as a response to a conspicuous behavior. This included making an effort to understand a student’s behavior by asking questions, or actively encouraging the respective student to open up after he or she was seeking someone to talk to. Surprisingly, the school counselor was involved in case assessment and management in only two cases. Furthermore, in 10 cases, schools responded to conspicuous behavior, or a behavior that has already been identified as warning behavior, with a pedagogical measure. This included light disciplinary measures (e.g., doing additional homework), the advice to cut back on a negative behavior to avoid severe consequences (e.g., as a response to aggressive tendencies), mediation, and conflict resolution (e.g., after a threat has been made in a conflict situation with a peer). In addition, conversations took place seeking to solve mainly academic problems instead of discussing a student’s concerns and problems at home (e.g., when a student’s academic performance dropped suddenly). In seven cases, stricter regulatory measures were implemented as a means to sanction the respective problem behavior (e.g., disciplinary difficulties). In four cases, a student was expelled from school or dismissed from vocational training after a major disciplinary issue (e.g., repeated or extended absence from school, student refused to follow instructions from school staff, or lacked compliance with legal guidelines). Regarding school-internal information exchange and case evaluation, we found that in eight cases, a school staff member chose to communicate the concerning observation to a colleague. However, our data reveal that a structured information exchange and assessment (e.g., the information was stored in a written document, or forwarded to the principal, and some criteria were applied to assess the behavior) was conducted in not more than two cases. Unfortunately, we were not able to obtain a proof of validity of the criteria or guidelines that were applied in these two cases and could not find any details about their origin in our material.

#### Professional Network Cooperation

In all 11 cases, a school staff member or the principal made an attempt to talk to a parent of the perpetrator (or vice versa) at least once, informing them about conspicuous behavior, a decline in academic performance, or disciplinary difficulties. This indicates that at some point during their negative developmental pathway, every perpetrator showed a behavior that urged school staff to establish cooperation with the families. However, further analysis revealed that this cooperation was often not sustained and intensified in the long term as no specific agreements or follow-up meetings were scheduled. In seven cases, schools forwarded a case to an external mental health institution for an in-depth diagnostic investigation or counseling or with the goal to send the student into long-term therapy (e.g., when noticing aggressive tendencies, social withdrawal, or suspecting family problems). Six students were hospitalized at some point during their psychosocial crisis, mostly short time for crisis intervention (e.g., after showing self-destructive tendencies or attempting suicide). The police got involved in four cases after a student made a threat providing concrete details about a potential offense. Surprisingly, despite being closely associated with the school context, school psychologists were only involved in three cases, and some witness reports point to an insufficient availability due to a lack of time resources.

### Research Question 2: Peer Responses to Threats and Leakage

We identified how peers responded to violence-specific, highly alarming warning behavior including threats, leakage, and having information that an attack was planned or knowing a perpetrator had access to a weapon. Estimating the effect of a peer response on the perpetrator’s motivation in terms of making him or her overthink or postpone the final decision to execute an attack is neither feasible nor useful in retrospect. Nevertheless, from a theoretical perspective, we found responses ranging from potentially facilitating an attack (e.g., making jokes or completely ignoring a threat) to probably averting an attack (e.g., discouraging the threatening peer from carrying out the original plan, or sharing information with others by seeking help from an adult). Table 3 reports the number of cases with reported peer responses.

**TABLE 3:** Peer responses to threats and leakage in school shooting cases in Germany.

**Responses**	**Number of cases**
Discourage perpetrator from executing the threat	9
Express concern, talk to perpetrator	8
Share information within peer group	7
Share information with an adult	5
Reduce contact or end relationship	5
Not take perpetrator seriously, laugh at perpetrator	4
Refused to help when asked	3
Incite perpetrator to start a shooting	3

In all 11 cases, at least one peer had some piece of knowledge about the perpetrator’s plans, or that he/she was considering a school shooting attack. In nine cases, a peer actively tried to discourage the perpetrator from executing a threat or leakage (e.g., by trying to minimize a perpetrator’s revenge fantasies or anger, highlighting positive aspects, or showing that one does not approve such behavior). In eight cases, a peer made an attempt to engage the perpetrator into a deeper conversation in order to obtain more information, learn more about a perpetrator’s actual motivation and struggles, and express their concern as a friend. Contrarily, being confronted with a threat, leakage, or planning behavior, in five cases, peers socially withdrew by reducing contact to the perpetrator, or ultimately ended the friendship. Peer reports reveal that either they were highly alarmed and anxious and tried to avoid the perpetrator entirely, or—in three cases—they did not expect the perpetrator to execute a threat or leakage. Nevertheless, peers were deeply irritated by the perpetrator’s behavior and could not identify with the friendship anymore. Furthermore, despite that some perpetrators leaked repeatedly, or made multiple threats, peers in four out of 11 cases reported retrospectively that they did not take these statements seriously, and—in three cases prior to reducing contact—laughed at the perpetrator and made jokes about a potential school shooting. In three cases, a peer was asked to help a perpetrator either in preparing an attack or serving as an accomplice in the attack. Simultaneously, in two of these cases and one other, other peers have reacted to the perpetrator’s violent plan by incitement (i.e., “go and do it”). Witness reports of the respective peers revealed, however, that none of them expected that the perpetrator would execute a school shooting and intended to use irony and jokes to “demonstrate the madness of the plan.” In seven cases, a concerned peer shared an observation within the peer group, discussing the probability of an attack. In five of these cases, code of silence was ignored and an observation or concern was reported to an adult (a teacher or parent).

### Research Question 3: Subjective Explanations and Factors Influencing the Identification of Warning Behavior

**FIGURE 1:**
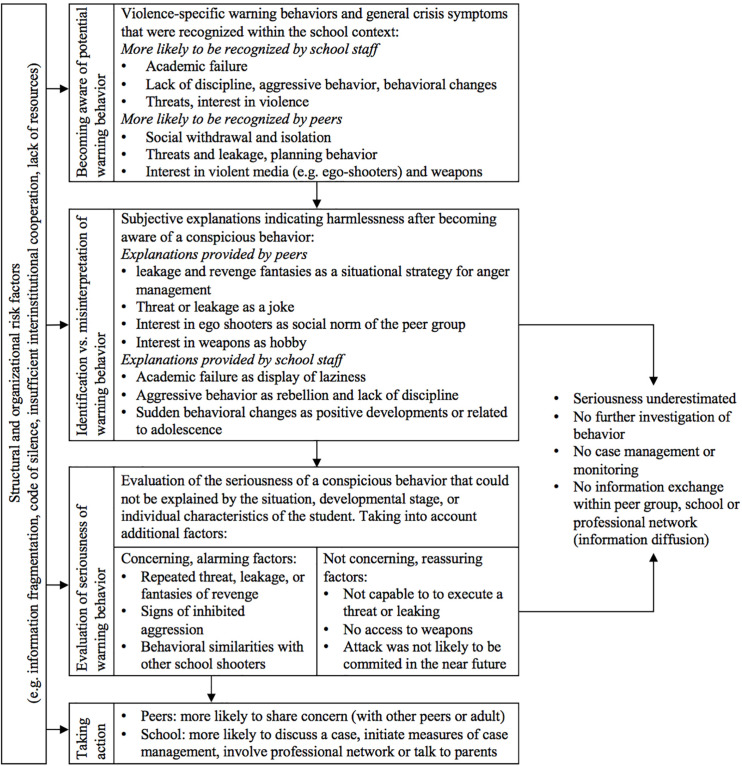
Subjective explanations and factors influencing the identification of warning behavior.

Witness reports from school staff and peers helped to understand why some behavioral changes of the perpetrators were identified as a warning behavior, or indicators for a psychosocial crisis, and others were ignored, trivialized, or not taken seriously within the school context. Thereby, we examined the following: (1) behavioral changes that were recognized in the first place and increased awareness of school staff and peers, (2) subjective explanations that school staff and peers gave to themselves to justify or contextualize a behavior, and how this led to information diffusion; (3) factors that were used to evaluate the seriousness of a behavioral change once it has been identified as warning behavior that could not be explained otherwise; and (4) action steps that were taken as a consequence of the seriousness evaluation. Figure 1 illustrates factors influencing the identification of warning behaviors recognized in 11 cases of school shootings in Germany.

#### Becoming Aware of a Conspicuous Behavior

School staff, in general, became aware of a student in crisis when the perpetrator made a direct threat or showed an interest in violence. *“On a Monday morning being asked about the past weekend, he talked about a movie he had seen, where a character was brutally thrown against a wall by an offender, and blood was running all over the place. He was fascinated by the fact that anyone could harm another person in such a way, and seemed to approve of this behavior. I had the impression he found a certain enjoyment in watching how others got tortured. All students in class could hear this, it was during third grade.”*

In addition to these violence-specific behaviors, school staff were naturally more likely to recognize indicators of a psychosocial crisis that became apparent in the classroom, were linked to academic success, or could not be ignored because the behavior disrupted daily routines (“The squeaky wheel gets the oil.”) as well as disciplinary difficulties, aggressive tendencies, and sudden behavioral changes. After learning from a colleague about a perpetrator’s conspicuous behavior in class, the school principal tried to obtain additional information: *“After hearing this, I had a closer look at the grades he got recently. I had to come to the conclusion that there in fact was a decline in important school subjects, which was definitely an information to be concerned about.”* As opposed to this, from all general crisis indicators, peers predominantly noticed social withdrawal and isolation, mostly referring to it as a well-known personality trait or tendency based on their experiences with the perpetrator. *“When I called him at home, he would respond immediately. I assumed that he rarely left the house to go out. He was the typical ‘quiet type of guy,’ almost invisible. I never thought he could do something like a school shooting.” “She was mostly online and glued to her computer all day. She didn’t want to engage in any activities with us.” or “He often was alone in the classroom, and didn’t have any real friendship with anyone.”* Additionally, peers became aware of violence-specific warning behavior, such as an interest in violent media, ego-shooters, and weapons. Furthermore, they were the primary recipients for leakage, planning behavior, or direct threats.

#### Subjective Explanations and Identification of a Warning Behavior

After becoming aware of a behavioral change, our data indicate that school staff as well as peers intuitively attempted to find explanations for an underlying motivation of the shown behavior. This includes explanations based on a situational dynamic, a perpetrator’s developmental stage, peer group norms, or other individual characteristics of the student. Moreover, differences are shown in the explanation for conspicuous behavior between peers and school staff. For instance, peers explained a leakage in combination with revenge fantasies with a perpetrator’s strategy to manage anger and regulate emotions after a conflict with a teacher. Being asked about a leakage made by a perpetrator during seventh grade, following a conflict with a teacher (“Someone should shoot him [the teacher] dead.”), a peer retrospectively reported: *“He always said this when he was angry at a specific teacher. I‘d say everyone has made such statements once in a while. That was not a plan nor an intention to be taken seriously.”* A similar situational explanation was provided by the peers during police investigation in another case, being asked if they never questioned the statements made by the perpetrator: *“Yes, we did ask him, why he would want to kill Ms M. He responded that he wants to kill her, because she said she wants to make sure he‘ll get expelled from school as soon as he gets a bad grade in a school report. According to him, she said this to him in person. He often had to stay behind after school when he didn‘t finish homework.”* Likewise, other leakages and even threats were interpreted as a joke by the peers, relying on a perpetrator’s non-verbal communication cues while making the statement (e.g., tone of voice, smiling), as the following quotations reveal: *“It‘s 3 weeks ago that he mentioned casually such a school shooting would be cool when we chatted about school shootings in general. But, he never said this in a serious tone. To me, it sounded like a joke so I didn‘t care about this a lot.” or “Well, yes, she was kind of interested in this [school shootings]. She also mentioned she wanted to do something like this, too. Anyway, there was always a touch of irony in her voice, so everyone of us thought she was making jokes or craving attention.”* A perpetrator’s interest in weapons or ego-shooter consumption was explained by a social peer group norm, which was socially approved and therefore not concerning: *“We knew he often played Counterstrike, we all did. He was pretty good at it. Everyone in school admired him for his gaming skills.”*

As opposed to this, school staff explained aggressive behavior as caused by rebellion and a lack of discipline, whereas academic failure was perceived to be a consequence of a perpetrator’s laziness instead of an indicator to a long-term critical development. *“By ‘popping up on the radar’ I mean that he showed rebellious and attention-seeking behavior. He refused to follow instructions from colleagues, and tried to get attention from classmates by giving childish answers to the teacher’s questions.”* Interestingly, school staff reported to have noticed sudden behavioral changes during the course of the negative development. While the majority of these new-quality behaviors were primarily alarming to teachers in the first place, most of them were eventually explained by typical adolescent behavior, and some were actually regarded as an indicator to a positive development. In one case, a perpetrator suddenly joined private tutoring—1 week before the shooting. *“His math teacher urged him to join tutoring for quite a while. However, he never showed any interest in following her recommendation, until last Tuesday, when he showed up for the very first time. We were positively surprised.”* Having a closer look at these results, we found that the availability of a subjective explanation for a conspicuous behavior generally indicated harmlessness and resulted in underestimating seriousness. An observation was consequently neither shared (e.g., within the peer group, school, or professional network) nor was a further investigation or case management procedure initiated, leading to information diffusion.

#### Evaluation of the Seriousness of a Warning Behavior

Only when no explanation was found, a conspicuous behavior was identified as a warning behavior, followed by an evaluation of its seriousness and—if concerning—a step to take action and respond. Our data revealed several additional factors that were taken into account to evaluate seriousness that were either alarming or reassuring. For instance, a once identified warning behavior was evaluated as non-serious when the perpetrator’s capability to execute a threat was doubted by the peers (*“He said he could never upset his parents, or harm them.”*), the perpetrator—presumably—did not have access to weapons, or the time span between a threat or leakage and a potential attack was overestimated. Being asked why he did not report his knowledge about a perpetrator planning an attack to the police, a peer responded: *[What made you think an attack would not happen too soon?] “Just because I didn‘t think she would be capable to carry out her threats. I didn‘t even consider she would prepare the Molotov cocktails on her own. Additionally, I found her problems rather manageable and comparatively small. That‘s why I never asked her about the threats.”* Consequently, the observation of the respective warning behavior was not shared or reported, leading to information loss. The perpetrator’s behavior was then neither monitored nor was help provided for him or her. As opposed to this, the repetition of a leakage or threat is mostly alarming, as well as when a perpetrator’s behavior was considered similar to a peer’s subjective assumptions about the behavior of a “typical school shooter”: according to peer statements in our data, this included violent media consumption, social isolation, or other conspicuous behavioral expressions. *“During the first times he said this, I just listened and thought by myself a lot of students claim this without ever carrying out a plan. It was, when he started to say these things repeatedly, and additionally mentioned to have an armed weapon at home, I told him to stop talking about this and such an idea would be complete nonsense.” [What led you and your friends to the conclusion that he was a “typical school shooter?”]. “He socially withdrew and was isolated. Apart from that, we knew he had all those weapons in his room and obsessively watched horror movies.”* Likewise alarming was an unexplainable conspicuous behavior that led to the impression that anger or aggression was inhibited and continued to grow below the surface. *“It was his way of speaking. There was a seriousness and certainty in his voice. I found him deeply agitated yet controlled. No loud yelling or venting his rage. When he said this, his voice was low-keyed. And he looked to the side with pure contempt.”* Eventually, if a warning behavior was accompanied by one of those factors, it was evaluated as serious, and further action was taken by sharing information with either other peers or—in some cases—an adult. Moreover, school staff discussed the respective case among colleagues, reported an observation to school officials, involved the professional network, talked to the parents, or initiated measures for case management.

## Discussion

The goal of the present study was to find answers to the question why past school shootings could not be averted by interventions from persons in a perpetrator’s school environment. Specifically, we aimed to understand why behavioral changes that retrospectively were identified as warning behaviors or symptoms of an individual crisis were not recognized, or—once noticed—were not evaluated as alarming signs. We focused our analysis on the initiated measures within the school as well as in cooperation with the student’s parents and professional network (research question 1). With regard to research question 2, we found peer’s reactions to alarming behavior to be varying in dependence of peer group norms as well as perceived seriousness of threats and leakage. When analyzing behavioral indicators that lead to an increased awareness of school staff and peers (research question 3), analysis revealed violence-specific behavior to be most likely recognized by school staff and peers. Regarding symptoms of psychosocial crisis, school staff mostly noticed academic failure and disrupting behavior in the class, while peers were more aware of the perpetrator’s social withdrawal and isolation. Interestingly also, the interpretation and evaluation of the identified indicators differed between school staff and peers. Peers misinterpreted threats or leakage as a joke or even understood the classmate’s anger toward a particular teacher. As a result, they underestimated the seriousness of the student’s intentions, also because they doubted the perpetrator’s capability of executing a threat. School staff explained sudden behavioral changes with typical adolescent behavior due to puberty or minor mental health issues. A closer evaluation and case management was only initiated if school staff or peers found no explanation at all for the perpetrator’s actions, identified “typical” indicators of a potential school shooter, or got the impression that the respective student kept anger hidden from their social environment.

As a starting point, we identified the measures and interventions that were initiated by school staff, as well as responses to warning behavior from peers. In all cases, school staff responded with measures within a school and contacted the student’s parent. Following this, we perused police witness reports for statements that provided insight into subjective explanations used by peers and teachers in an attempt to make sense of a conspicuous behavior. Furthermore, we extracted a list of alarming and reassuring factors that were considered when evaluating a conspicuous behavior. First, our data revealed that in a large number of occasions when a warning behavior was adequately identified, a perpetrator’s social system responded appropriately. In all 11 cases, at some point along the negative psychosocial pathway, a school staff member or peer engaged in a one-on-one conversation with the perpetrator, as well as collaborated with the parents initiating a conversation about him or her. Moreover, for a variety of reasons—not necessarily based on the assumption of a school shooting—almost all cases were referred to counseling, diagnostic investigation, or therapy, or talked to a school psychologist at least once. However, despite these measures, none of the perpetrators was stopped from executing his/her attack. Data showed that students’ decline in grades or violence-related behavior often resulted in sanctions instead of resource-orientated support to prevent an escalation of the student’s psychosocial crisis. This result stands in line with research on US-American cases in which school suspensions and expulsions reinforced the student’s crisis that escalated toward the violent act (cf. Borum et al., 2010; Muschert and Madfis, 2013). Our analysis revealed that—in retrospect—these behaviors were symptoms of a negative psychosocial development, and school officials and staff did not have information about a student’s trouble (e.g., family or social problems) and insufficient coping mechanisms. Following this, an isolated conspicuous or threatening behavior was—probably due to structural barriers and a lack of knowledge—evaluated without taking the whole picture into account. This ultimately resulted in responses that were more likely fueled by an understandable fear rather than coming from a perspective of preventing a negative psychosocial development. Research on developmental pathways toward school shootings gives evidence that perpetrators made efforts to cope with their psychosocial strain that accompanied the planning and fantasizing about the later violent act by opening up and communicating their problems to others (Sommer et al., 2020). These often hidden and ambiguous attempts of functional coping were mostly interpreted as a reflection of the perpetrator’s positive development but consequently went unnoticed or unacknowledged by school staff or peers.

In the majority of cases, violence-specific behavior, such as threats, leakage, and planning behavior, induced concern or even fear in both school staff and peers alike. As opposed to this, general crisis symptoms that were more subtle or hidden (e.g., depressive tendencies and social withdrawal) were not given much attention. This is in line with studies from Fox and Harding (2005) that pointed to the “decoy problem” in large organizations. Students, or perpetrators, respectively, who disrupted a school’s daily routine by showing aggressive behavior, or became apparent through academic failure or school distance—all being familiar behaviors to school staff and easily recognizable in the classroom—popped up on the radar, which resulted in a deeper investigation or a pedagogical reaction (“The squeaky wheel gets the oil.”). While this perceptive bias generally ensures an organization’s day-to-day functioning, it can result in overseeing more “invisible” warning behaviors of quieter students (Fox and Harding, 2005). Bender et al. (2001) refer to these “Invisible Kids” in an article highlighting the importance of paying attention to students that internalize anger and, thus, will more likely display subtle behavioral changes (e.g., depressive symptoms, self-destructive tendencies, and emotional withdrawal) instead of overt aggression, even when in severe crisis. Furthermore, our data revealed that school staff as well as peers relatively quickly attempted to find subjective explanations for a concerning behavior, which ultimately resulted in minimizing or trivializing it. While in general, we would encourage a perspective on adolescent behavior that is not based on fear as it can lead to stigmatization or exclusion of individual students (Borum et al., 2010), we assume that some warning behaviors would have been evaluated as alarming, if more background information about a student’s situation had been available. Since our funding resources, as well as methodological considerations (e.g., retrospective bias), did not allow the conduct of in-depth interviews with officials from the schools where a shooting happened, we can only rely on witness reports when making assumptions about institutional information loss, i.e., information fragmentation and information diffusion that were found in a United States case study (Fox and Harding, 2005). Whereas we found various indicators to information exchange between two school staff members in 10 cases, these conversations in at least nine cases did not follow a structured protocol and were most often not documented or elaborated in a larger team-based case discussion. To sum up, not knowing if any other warning behaviors had been previously identified resulted in underestimating the seriousness of a conspicuous behavior, and the respective behavior was therefore not documented or exchanged within a school’s professional network or among school staff and peers, respectively.

As previously found in US-American case studies, peer “code of silence” additionally fostered information fragmentation in the German cases. The result that in only five cases the classmate’s conspicuous behavior was reported to an adult stands in line with research by Pollack et al. (2008) as well as Madfis (2014), discussing variables that influence the bystanders’ decision to reveal information to school staff regarding threats. Those students who were unwilling to come forward indicated that they anticipated getting into trouble or being interrogated if they share certain information (Pollack et al., 2008), leading to the general finding that positive relations with teachers or other adults serve as a necessary condition for breaking the “code of silence.” Moreover, a study focusing on averted rampage attacks suggests that school shootings might, at least in part, be prevented if the school’s culture is sufficiently positive for students to feel comfortable telling school staff about conspicuous behavior. Similar to our results, in cases of averted school shootings, bystanders neglected to come forward with threatening behavior because they lacked certainty about the respective student’s intentions or interpreted threats and leakage as innocent comments or jokes (Madfis, 2014).

Moreover, in some cases, the lack of knowledge about the perpetrator’s problems, as well as early yet unspecific indicators of a personal crisis, resulted in overly strict disciplinary measures when—in retrospect—measures for student support would have been more appropriate for intervention in order to prevent an individual crisis (Borum et al., 2010). Finally, our data revealed that most schools at that time did not have specific guidelines for case management and only involved school psychologists or other counseling experts relatively late in an attempt to stick to their own resources and expertise. This result stands in line with research on cases of psychosocial crisis potentially leading to school shootings (Sommer et al., 2016), in which school staff even in cases of high risk (e.g., access to weapons and repeated threats) preferred to initiate measures within the school routine instead of collaborating with the professional network. It can be only hypothesized that school staff hesitated to involve external stakeholders to preserve the student from stigmatization and avoid reputational damage to the school. Furthermore, in cases that were subject to structured assessment, a diagnostic investigation of mental health problems, or even temporary hospitalization, our data indicate that experts were not sufficiently trained in threat assessment and the handling violence-specific warning behavior, e.g., revenge fantasies. Furthermore, schools and other institutions involved in case management were generally not aware of the importance of long-term case monitoring. Mostly, after a first evaluation and primary response to a warning behavior, a case was considered completed, without further inquiry into the effectiveness of the intervention measures or a monitoring of the perpetrator’s psychosocial development. This, for instance, even resulted in misinterpreting a—retrospectively—conspicuous behavior shortly before the attack as an indicator to a positive development of the student.

### Limitations

Our case data are based on witness reports obtained during police investigation and therefore carry some inherent methodological limitations that need to be considered carefully. First, our findings rely on the subjective, retrospective perspective of peers and school staff who still might have been emotionally or cognitively influenced at the time of police investigation—even weeks after the attack. Especially for those peers or teachers emotionally involved with the perpetrator, trauma-induced anxiety, memory gaps, or cognitive distortion together with a subjective fear of legal consequences or irrational feelings of guilt might have ended up in witness statements that differ from what someone would answer when being asked by a scientific interviewer. Moreover, it can be assumed that being asked about the perpetrator’s development and conspicuous behavior induced high levels of stress, which is found to negatively affect both accuracy of eyewitness identification as well as accuracy of recall of crime-related details (Deffenbacher et al., 2004). While it is certain that police investigation was only conducted when a witness voluntarily joined, and appeared to be mentally and emotionally stable and accountable, we cannot deny the potentially traumatizing impact of an act of school shootings on survivors. More specifically, we can only roughly imagine the thoughts and feelings of a peer who had some information prior to the attack, but did not report it, or of a teacher who had some conflict with a perpetrator. On top of that, since the primary purpose of police investigation after an attack is to investigate motives, reasons, and responsibilities, it is possible that, when being asked about warning behaviors, witnesses naturally attempt to minimize an alarming behavioral change or deny a bad gut feeling retrospectively. Moreover, when analyzing rare events such as school shootings, we must take into account the low generalizability of findings when making assumptions about the “population” of school shooters or when drawing general conclusions for prevention (for an overview, see Harding et al., 2002). The deduction of sufficient conditions for the identification, evaluation, and management of conspicuous behavior is dependent on the comparison of our cases with averted violent acts (cf. Madfis, 2014). Thus, our results are only valid for those 11 cases under study and, therefore, fail in transferability to similar objects of investigation from other countries.

### Implications and Outlook

Based on what we know today after an attack has happened, and following an intense case study, we can draw the conclusion that the majority of warning behaviors were misinterpreted or their seriousness underestimated due to insufficient communication about the student within the school and within the professional network (i.e., information fragmentation). Additionally, peer code of silence, a lack of knowledge about early unspecific indicators of a negative psychosocial development, and a lack of structured procedures for information exchange and documentation (i.e., information diffusion) as well as for effective case management including a long-term monitoring resulted in unspecific responses to violence-specific warning behaviors (i.e., threats or leakage). More broadly, persons in the school environment of a perpetrator did not have sufficient knowledge, information, and official guidelines that allowed them to simultaneously take violence-specific warning behaviors, general crisis symptoms, and a student’s overall situation into account in order to draw conclusions about a negative psychosocial development. These findings, while certainly limited to our sample, however, point to opportunities of prevention when being taken into account together with results on organizational deviance and institutional information loss. An empirically based preventive intervention should include strategies to ensure that (1) school staff is trained to recognize a student crisis based on indicators that are not limited to academic and disciplinary difficulties, or alarming violence-specific behaviors; (2) an individual teacher or peer shares a bad gut feeling with at least one other person, even when he or she initially doubts its significance; (3) schools as complex organizations should follow a formal protocol that includes reliable criteria when evaluating the seriousness of a warning behavior, e.g., a direct threat, and take various perspectives into account; and (4) schools should establish a procedure for case management and involve partners from their professional network more often and early enough. To sum up, based on our findings, we recommend the implementation of an organizational structure that allows a person who observes a warning behavior to easily access knowledge about the student’s background, as well as to share an observation and obtain information on warning behaviors that were previously observed by others. This would most probably result in a more accurate evaluation of the seriousness of a concerning behavior as well as increase a person’s willingness to report a warning behavior—even when it does not appear to be alarming in the first place. Additionally, peers can help to avert school shootings if they become aware of a leakage, threat, or other warning behavior and are encouraged to break the code of silence and preferably “over-share” a concern or uncertainty (e.g., through an open school climate, or the opportunity to report anonymously) (Pollack et al., 2008; Eliot et al., 2010; Madfis, 2014). Finally, our analysis revealed an increased need for expert knowledge and skills when dealing with students who show violence-specific warning behavior or are suspected to engage in planning behavior. To our view, providing mental health professionals, as well as school psychologists with specific guidelines regarding the diagnostic investigation and treatment of adolescents who repeatedly communicate threats, violent fantasies, or leakage would increase effectiveness and sustainability of a professional, community-based case management. As the purpose of qualitative research is not to provide generalizable findings, the results of our study may serve as a foundation for future quantitative or mixed-methods studies. However, by using the chosen approach, we introduced and described new emerging concepts such as the subjective explanations of conspicuous behavior, which were not visible to us beforehand and have not yet been a subject of investigation in this detail. To our knowledge, the present study is the first investigation focusing on responses by school staff and peers in a sample of German cases of school shootings. Future research should put an emphasis on cross-national differences as well as intercultural influences when examining the relevance of a perpetrator’s social environment in averting school shootings.

## Data Availability Statement

The datasets generated for this study will not be made publicly available. The datasets analyzed for this study, i.e., original inquiry files from law enforcement authorities, cannot be shared for legal and privacy restrictions.

## Author Contributions

HS and VL contributed to the conception and design of the study. NF, KG, NA, and FS organized the database. NF performed the qualitative analysis and wrote the first draft of the manuscript. FS, VL, NA, KG, and HS wrote sections of the manuscript. All authors contributed to manuscript revision and read and approved the submitted version.

## Conflict of Interest

The authors declare that the research was conducted in the absence of any commercial or financial relationships that could be construed as a potential conflict of interest.
